# Impact of atorvastatin and mesenchymal stem cells combined with ivermectin on murine trichinellosis

**DOI:** 10.1007/s00436-023-08077-x

**Published:** 2023-12-18

**Authors:** Zeinab R. Hassan, Samar El-Sayed, Kareman M. Zekry, Samah Gouda Ahmed, Asmaa Hassan Abd-Elhamid, Doaa E. A. Salama, Azza Kamal Taha, Nihal A. Mahmoud, Shaymaa Fathy Mohammed, Mona M. Amin, Rasha Elsayed Mohamed, Ayat M. S. Eraque, Shimaa A. Mohamed, Ranya M. Abdelgalil, Shimaa Attia Atta, Nermeen Talaat Fahmy, Mohamed S. Badr

**Affiliations:** 1 Department of Parasitology, Faculty of Medicine for Girls, Al-Azhar University, Yosief Abbas Street, Cairo, Kairo, Egypt; 2 Department of Histology, Faculty of Medicine for Girls, Al-Azhar University, Yosief Abbas Street, Cairo, Kairo, Egypt; 3 Department of Pathology, Faculty of Medicine for Girls, Al-Azhar University, Yosief Abbas Street, Cairo, Kairo, Egypt; 4 Department of Physiology, Faculty of Medicine for Girls, Al-Azhar University, Yosief Abbas Street, Cairo, Kairo, Egypt; 5 Department of Pharmacology, Faculty of Medicine for Girls, Al-Azhar University, Yosief Abbas Street, Cairo, Kairo, Egypt; 6 Department of Biochemistry, Faculty of Medicine for Girls, Al-Azhar University, Yosief Abbas Street, Cairo, Kairo, Egypt; 7 Department of Anatomy and Embryology, Faculty of Medicine for Girls, Al-Azhar University, Yosief Abbas Street, Cairo, Kairo, Egypt; 8 Department of Immunology, Theodor Bilharz Research Institute, 36VF+MJ2, Warraq Al Arab, El Warraq, Giza Governorate, 3863130 Egypt; 9 Genomics, Egypt Center for Research and Regenerative Medicine (ECRRM), 3 Emtedad Ramses, Al Abbaseyah Al Gharbeyah, El Weili, Cairo Governorate, 4435102 Egypt; 10 Molecular Biology and Genetic-Bioinformatics Nano-Robot Diagnostics, Medical Research Centre, Faculty of Medicine, Ain Shams University, El-Khalyfa El-Mamoun Street Abbasya, Cairo, Egypt; 11 Department of Pathology, School of Medicine, Badr University in Cairo (BUC), Entertainment Area, Badr City, Cairo, 11829 Egypt

**Keywords:** Angiogenesis, Atorvastatin, CD31, Mesenchymal stem cells, Ivermectin, Trichinellosis, Antioxidant

## Abstract

Trichinellosis is one of the global food-borne parasitic diseases that can cause severe tissue damage. The traditionally used drugs for the treatment of trichinellosis have limited efficacy against the encysted larvae in the muscular phase of the disease. Therefore, this study aimed to evaluate the role of atorvastatin and mesenchymal stem cells combined with ivermectin against different phases of *Trichinella* in experimentally infected mice. A total of 120 male Swiss albino mice were divided into two major groups (*n* = 60 of each), intestinal and muscular phases. Then, each group was subdivided into 10 subgroups (*n* = 6); non-infected control, infected non-treated control, infected ivermectin treated, infected atorvastatin treated, infected mesenchymal stem cells treated, infected combined ivermectin and atorvastatin treated, infected combined mesenchymal stem cells and ivermectin treated, infected combined mesenchymal stem cells and atorvastatin treated, infected combined mesenchymal stem cells and a full dose of (ivermectin and atorvastatin) treated, and infected combined mesenchymal stem cells and half dose of (ivermectin and atorvastatin) treated. Mice were sacrificed at days 5 and 35 post-infection for the intestinal and muscular phases, respectively. The assessment was performed through many parameters, including counting the adult intestinal worms and muscular encysted larvae, besides histopathological examination of the underlying tissues. Moreover, a biochemical assay for the inflammatory and oxidative stress marker levels was conducted. In addition, levels of immunohistochemical CD31 and VEGF gene expression as markers of angiogenesis during the muscular phase were investigated. The combined mesenchymal stem cells and atorvastatin added to ivermectin showed the highest significant reduction in adult worms and encysted larvae counts, the most noticeable improvement of the histopathological changes, the most potent anti-inflammatory (lowest level of IL-17) and anti-angiogenic (lowest expression of CD31 and VEGF) activities, and also revealed the highly effective one to relieve the oxidative stress (lowest level of SOD, GSH, and lipid peroxidase enzymes). These observed outcomes indicate that adding mesenchymal stem cells and atorvastatin to ivermectin synergistically potentiates its therapeutic efficacy and provides a promising candidate against trichinellosis.

## Introduction

Trichinellosis is a parasitic zoonosis that is distributed globally and is commonly caused by *Trichinella spiralis (T. spiralis)* (Rainova et al. 2016). Human infection usually occurs after consuming raw or undercooked pork meat containing encysted larvae of *T. spiralis* (Gottstein et al. 2009). During the first week after infection, the intestinal phase of trichinellosis occurs due to the colonization of the small intestine by the adult stages of the parasite resulting in damage of the intestinal villi that is evident by fever, nausea, vomiting, and diarrhea. The adult female worms produce larvae that penetrate the striated muscles during the muscular phase and form the nurse cell complexes surrounded by collagen capsules causing manifestations of fever, myalgia, muscle weakness, and periorbital edema (Ren et al. 2013). The parasite forms this nurse cell complex to protect itself against the host’s immune reaction and to take nourishment via eliciting the angiogenesis to recruit new blood vessels in the collagen capsules that are synthesized by the nurse cell and mainly consist of collagen IV and VI types. In this concern, nurse cell complexes stimulate the vascular endothelial growth factor (VEGF) as a potent mediator for the angiogenic process (Ock et al. 2013).

Benzimidazole derivatives (e.g., mebendazole and albendazole) are the traditional treatment for trichinellosis (Gottstein et al. 2009). In addition, several studies reported the activity of ivermectin (IVM) against *T. spiralis* (Basyoni and El-Sabaa 2013). However, these drugs have limited effect against the encapsulated larvae besides their low bioavailability (Yadav and Temjenmongla 2012; Hassan et al. 2021). Statins, e.g., atorvastatin, have a hypolipidemic activity in atherosclerosis and coronary artery diseases (Profumo et al. 2014). Furthermore, atorvastatin has demonstrated activity against different phases of trichinellosis through its antioxidant, anti-inflammatory, and anti-angiogenic effects (Othman et al. 2016). On the other hand, mesenchymal stem cells (MSCs) can contribute to tissue regeneration physiologically or even after exposure to damage (Osakada et al. 2010). Besides their regenerative potentiality, they have immunomodulatory effects, and due to these properties, MSCs have been used to treat different parasitic diseases such as leishmaniasis, trypanosomiasis, schistosomiasis, and hydatid disease (Rayia et al. 2017; Aryamand et al. 2017; Abo-Aziza et al. 2019). Furthermore, MSCs have demonstrated potent anti-*Trichinella* activity when added to standard chemotherapeutic drugs (Sarhan et al. 2021). Therefore, the present work aimed to study the impact of combined atorvastatin and MSCs when added to ivermectin on experimental trichinosis in murine models.

## Materials and methods

**Diagram 1:**
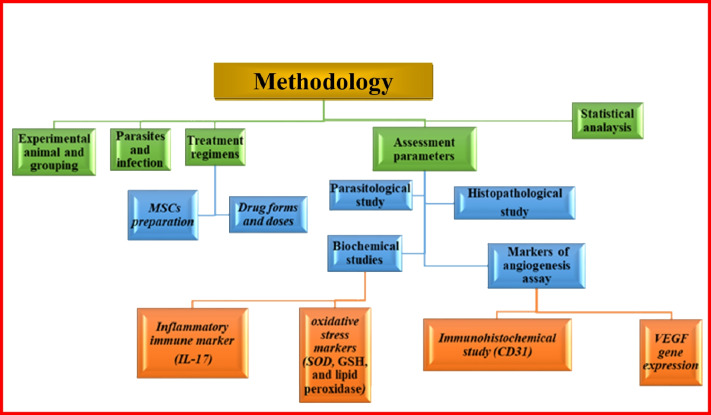
Scheme of methodology design

This experimental study (Diagram 1) was conducted at Theodor Bilharz Research Institute (TBRI) and Parasitology Department, Faculty of Medicine for Girls (FMG), Al-Azhar University, in cooperation with the Molecular Research Center, Faculty of Medicine, Ain Shams University, Egypt.

### Experimental animals and grouping

**Diagram 2:**
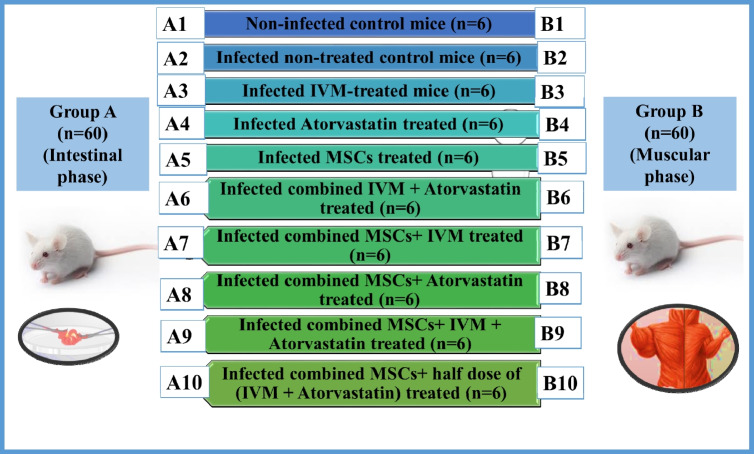
Animal groups and their characteristics

The present work was carried out on 120 laboratory-bred Swiss albino male mice aged 4–5 weeks and weighing 20–25 g. They were pathogen-free, provided by TBRI, and housed at suitable food, temperature, and water supply according to laboratory standards. The mice were divided into two groups (60 mice each); Group A represents the parasite’s intestinal phase, and Group B represents the muscular phase. Then, each group was subdivided into 10 subgroups (6 mice each) (Diagram 2). Mice of the intestinal phase were sacrificed on the 5th day post-infection (PI), while those of the muscular phase were sacrificed on the 35th day PI. Sacrification was performed under intraperitoneal anesthesia, according to Liang et al. (1987). The collected samples from the two phases underwent further assessment through parasitological and histopathological studies. Serological and tissue biochemical assays, as well as evaluation expression of angiogenesis markers, were conducted.

#### Parasites and infection

*T. spiralis* larvae were obtained from the maintained successive cycles at TBRI. The infected mice were sacrificed and dissected to obtain the infected muscles that were further digested and minced by incubation for 2 h at 37 °C in 1% pepsin-hydrochloride added to distilled water. The digested mixture was filtrated and washed several times in distilled water, then allowed to sediment to collect the free larvae. Each mouse was orally infected with nearly 200 living motile larvae (Othman et al. 2016).

#### Treatment regimens

The treatment was administrated on day 1 PI in the intestinal phase and day 14 PI in the muscular phase. In the two phases, both IVM and MSCS were administrated as a single dose (Etewa et al. 2019; Hassan et al. 2021), while atorvastatin was administrated for 5 consecutive days (Madbouly Taha et al. 2017; Basyoni et al. 2018).

##### MSCs preparation

The used MSCs were derived from the bone marrow by collecting femur and tibia bones and flushing out the bone marrow of laboratory-bred mice. Isolate bone marrow cells were isolated by centrifuging the cell suspension and resuspending the pellet in a complete culture medium (Dulbecco’s Modified Eagle Medium, “DMEM,” Gibco, UK). Then, the cells were cultured by placing them in tissue culture flasks with a complete culture medium supplemented with fetal bovine serum and antibiotics (Gibco, UK) and replacing the medium every 2–3 days to maintain the cell attachment and growth. The cells were passaged when they reached 70–90% confluence by detaching them with trypsin–EDTA solution (Gibco, UK) and subculturing them in new flasks (NUNC Company, Denmark). The presence of MSCs was confirmed by performing phenotypic analysis using flow cytometry, looking for MSC CD29, CD44, and CD73 markers (Dako, USA) (Pulavendran et al. 2010). Trypan blue exclusion assay was conducted to count the number of viable cells, then mice at the intestinal phase (Group A5, A7, A8, A9, and A10) and muscular phase (Group B5, B7, B8, B9, and B10) were injected intraperitoneally by 1,000,000 of viable cells/mouse using an insulin syringe (Etewa et al. 2019; Sarhan et al. 2021).

##### Drug forms and doses

IVM (Iverzine, UNI Pharma, Cairo, Egypt) and atorvastatin (Ator, AUG Pharma, Cairo, Egypt) tablets were ground, dissolved freshly in distilled water, and then administrated orally. At both intestinal and muscular phases, IVM was given at a dose of 0.2 mg/kg (as a full dose) and 0.1 mg/kg (as a half dose), while atorvastatin was given at a dose of 40 mg/kg/day (as a full dose) and 20 mg/kg/day (as a half dose) (Basyoni et al. 2018; Hassan et al. 2021).

### Assessment parameters

#### Parasitological study

##### Intestinal phase

The small intestines of the infected mice were removed (on day 5 PI) and dissected longitudinally, then further processed according to Salama et al. (2022), to collect the intestinal fluid that was examined to detect and count the adult *T. spiralis* worm burden/milliliter under a dissecting microscope.

##### Muscular phase

The muscles of the infected mice were dissected (on day 35 PI), weighed, divided into small parts then digested and centrifugated. The sediment was examined microscopically to detect and count the larvae burden/gram muscle tissue (Sarhan et al. 2021).

#### Histopathological study

The excised small intestine (from the intestinal phase) and muscle specimens (from the muscular phase) were fixed immediately in 10% formol-saline, then further subjected to paraffin embedding procedure and 4 µm thickness were cut and stained with Hematoxylin & Eosin stain (H & E) to be examined microscopically (Shalaby et al. 2010).

#### Biochemical studies

##### Assessment of the inflammatory immune marker in the serum

The collected blood samples from the mice of the intestinal (at day 5 PI) and muscular (at day 35 PI) phases were left to clot for 20 min at room temperature and then centrifuged at 2000 rpm for 10 min. The sera were aliquoted and stored at − 20 °C till used. Levels of IL-17 were estimated by ELISA using commercially available kits (SUNLONG, China). In brief, serum samples were incubated with the immobilized specific antibodies and visualized using an HRP-TMB reaction. According to the manufacturer’s instructions, the total concentrations were measured at an optical density of 450 nm, then calculated using the standard curve (Hafez et al. 2021).

##### Assessment of the oxidative stress markers in the tissue homogenates

Using an automated homogenizer, 1 gm of intestinal (at day 5 PI) and muscular (at day 35 PI) 0.5 gm of tissues were homogenized in 10 ml PBS. Each homogenate was filtered via filter paper, and the filtrate was used for the assessment of oxidative stress markers (superoxide dismutase (SOD), glutathione (GSH), and lipid peroxidase) using commercially available kits (Biodiagnostics, Egypt). The total concentrations were calculated using the Bradford assay. Units are expressed in U/ml tissue homogenate (Bradford 1976; Hafez et al. 2021).

#### Markers of angiogenesis assay

##### Immunohistochemical study

The microvessel density (MVD) proliferation was evaluated by measuring the average number of CD31 positive-stained endothelial cells. Paraffin sections with 4 µm thickness from the tongue of mice at the muscular phase (Groups B1–B10) were mounted on electrically positive charged glass slides for the further immune staining procedure according to Cury et al. (2020) and Rayia et al. (2022) using CD31 monoclonal antibody (Cell Marque, Rocklin, CA, USA; clone JC70, mouse monoclonal) at 1:100 dilutions. Standard negative and positive control sections were used. Further evaluation was performed by two expert pathologists by counting CD31 positive luminal structures in each slide under the light microscope. Based on a brown membranous cytoplasmic stain, the intensity and percentage of the staining of inflammatory cells were calculated according to Cury et al. (2020), then MVD was scored as weak (< 10% stained cells), medium (10–20%), and strong (20–45%).

##### Molecular VEGF gene expression assay by real-time PCR

Fresh muscle specimens (at day 35 PI) were homogenized and preceded to RNA isolation according to the manufacturer’s protocol of RNeasy Mini Kit (Qiagen). The extracted RNA’s reverse transcription was conducted using a high-capacity cDNA transcription kit (Applied Biosystems). Each product was subjected to a real-time PCR assay, and glyceraldehyde 3-phosphate dehydrogenase (GAPDH) was used as an internal control. The primers used for VEGF were 5′-CTTGCCTTGCTGCTCTACC-3′ and 5′-CACACAGGATGGCTTGAAG-3′, and those for GAPDH were 5′-CTCATGACCACAGTCCATGC-3′ and 5′-TTCAGCTCTGG GATGACCTT-3′. The reaction was performed according to the standard protocol of 7500-Applied Biosystems via a thermal cycling program as the following; 95 °C for 10 min, then 95 °C for 15 s, and 60 °C for 1 min. The VEGF expression levels were calculated using the 2^−ΔΔCt^ method according to the manufacturer’s recommendations (Schmittgen and Livak 2008; Khositharattanakool et al. 2013).

### Statistical analysis

The collected data were analyzed and evaluated by the Statistical Package for Social Sciences (SPSS) program, version 22, then expressed as mean and standard deviation (SD). The possible statistical differences between groups were determined using the student *t*-test and analysis of variance (ANOVA) *f*-test. The significant level was considered at a *p-*value of < 0.05. The percentage of reduction (*R*%) was calculated using the formula (*R*%) = [(*A* − *B*)/*A*] × 100, where *A* is the mean count of the infected non-treated group, and *B* is the mean count of the infected-treated group.

## Results

### Parasitological study results

#### Parasitological intestinal phase results

A significant reduction in the mean count of the adult worms was noticed among the infected treated intestinal phase (Groups A3–A10) compared to the infected non-treated control (Group A2) at day 5 PI (Table 1). In addition, infected mice treated with the combined full dose of IVM and atorvastatin added to MSCs (Group A9) exerted the highest percentage of reduction, followed by infected mice treated with a combined half dose of IVM and atorvastatin added to MSCs (Group A10). In contrast, the lowest reduction percentage was observed with infected mice treated with atorvastatin monotherapy (Group A4) (Table 1).

**Table 1:** Intestinal phase mean counts and percentages of reduction of the adult* T. spiralis* worms

Groups	Number of *T. spiralis* adults/ml of the intestinal fluid		
	Mean ± SD	*T*-test with group A2 (*p*-value)	Reduction (%)
A2	77.33 ± 7.5		
A3	21.33 ± 3.06	*P* < 0.001*	72.4%
A4	47.67 ± 4.04	*P* < 0.001*	38.4%
A5	36.33 ± 4.04	*P* < 0.001*	53.02%
A6	19.67 ± 2.08	*P* < 0.001*	74.6%
A7	15.33 ± 2.08	*P* < 0.001*	80.2%
A8	35.33 ± 4.51	*P* < 0.001*	54.3%
A9	10.33 ± 1.53	*P* < 0.001*	87.6%
A10	13.67 ± 7.24	*P* < 0.001*	82.3%
*F*-test	135.96		
*P*-value	0.000*		

Group A2: infected non-treated control mice

Group A3: infected IVM-treated mice

Group A4: infected atorvastatin-treated

Group A5: infected MSC-treated

Group A6: infected combined IVM + atorvastatin

Group A7: infected combined MSCs + IVM

Group A8: infected combined MSCs + atorvastatin

Group A9: infected combined MSCs + IVM + atorvastatin

Group A10: combined MSCs + half dose of (IVM + atorvastatin)

^*^P < 0.05

#### Parasitological muscular phase results

At day 35 PI, it was observed that all infected treated muscular phase groups (B3–B10) resulted in a significant reduction in the mean count of the encysted larvae compared to the infected non-treated control (Group B2) (Table 2). Regarding the percentage of reduction, it was highest with infected mice treated with the combined full dose of IVM and atorvastatin added to MSCs (Group B9), followed by infected mice treated with a combined half dose of IVM and atorvastatin added to MSCs (Group B10). Conversely, the infected mice treated with atorvastatin monotherapy (Group B4) revealed the lowest reduction percentage (Table 2).

**Table 2:** Muscular phase mean counts and percentages of reduction of the *T. spiralis* larvae

Groups	Number of *T. spiralis* larvae/1gm of the muscular tissue		
	Mean ± SD	*T*-test with group A2 ( *p*-value)	Reduction (%)
B2	116.33 ± 7.02		
B3	24.67 ± 2.51	*P* < 0.001*	78.8%
B4	63.67 ± 4.16	*P* < 0.001*	45.3%
B5	50.33 ± 3.51	*P* < 0.001*	56.7%
B6	24.33 ± 2.52	*P* < 0.001*	79.1%
B7	22.67 ± 1.53	*P* < 0.001*	80.5%
B8	37.67 ± 5.03	*P* < 0.001*	67.6%
B9	18.67 ± 3.05	*P* < 0.001*	83.95%
B10	20.67 ± 2.08	*P* < 0.001*	82.2%
*F*-test	410		
*P*-value	0.000*		

Group B2: infected non-treated control mice

Group B3: infected IVM-treated mice

Group B4: infected atorvastatin-treated

Group B5: infected MSC-treated

Group B6: infected combined IVM + atorvastatin

Group B7: infected combined MSCs + IVM

Group B8: infected combined MSCs + atorvastatin

Group B9: infected combined MSCs + IVM + atorvastatin

Group B10: infected combined half dose of MSCs + half dose of (IVM + atorvastatin)

^*^P < 0.05

### Histopathological results

#### Histopathological intestinal phase results

**Fig. 1:**
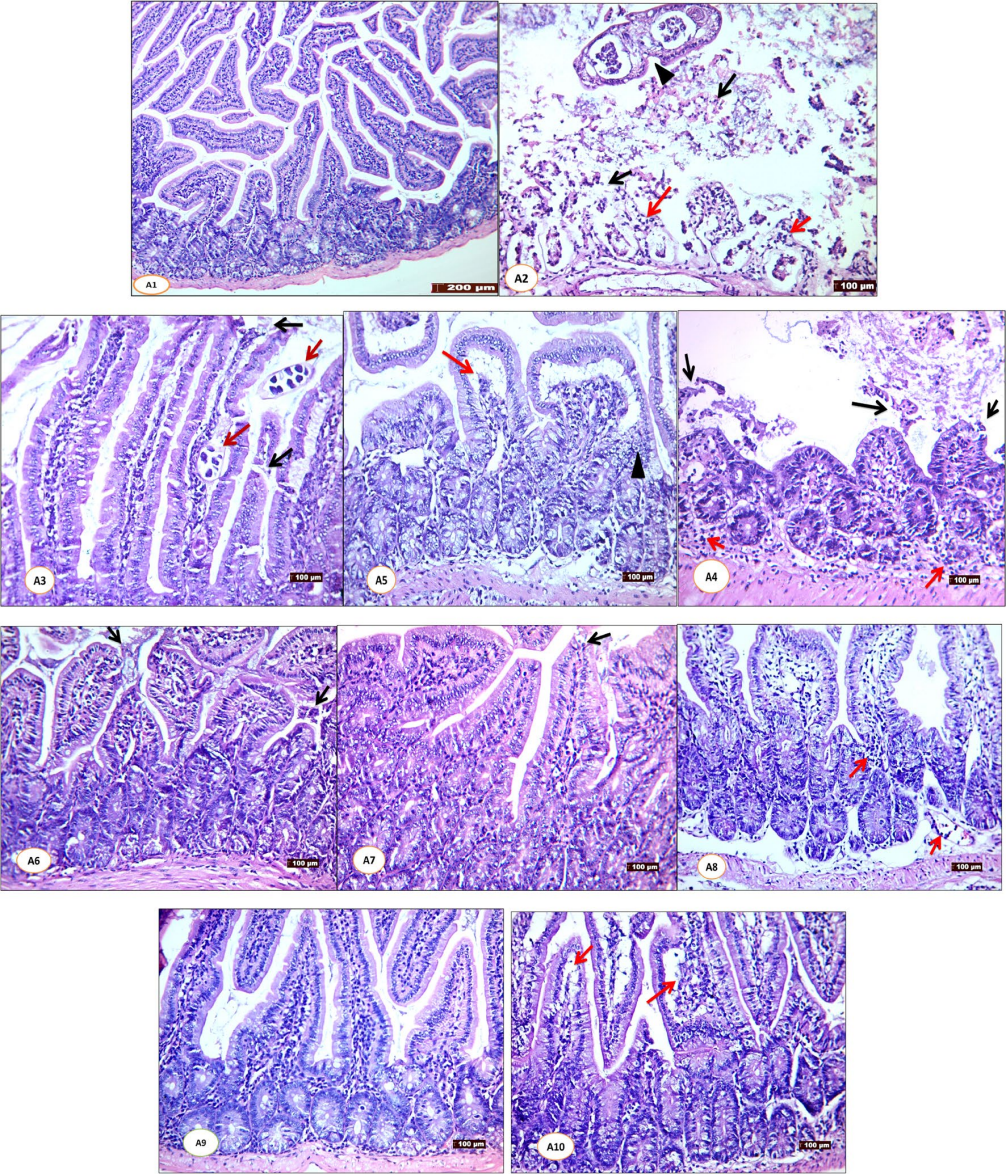
Sections of small intestine stained with H&E from studied groups (**A1**–**A10**) on the day 5 PI; **A1** (non-infected control group) showing the normal architecture of intestinal villi and crypts which appear long, regular, and lined by simple columnar epithelium with goblet cells with a core of connective tissue (no histopathological changes) (100 ×). **A2** (infected non-treated control group) showing sloughing and distortion of the villi (black arrows), cut sections in *T. spiralis* adults appear in the lumen and the core of villi (arrowhead), dense inflammatory cellular infiltrate (red arrows) (100 ×). **A3** (Infected IVM-treated group) (200 ×) showing cut sections in *T. spiralis* adults appear in the core of the villi and the lumen (red arrows) and sloughing of the tip of some villi (black arrows) in **A4** (infected atorvastatin-treated group) showing shortening of the villi with sloughing of its tip (black arrows) and moderate inflammatory cellular infiltrate (red arrows) (200 ×). **A5** (Infected MSC-treated group) showing vacuolation of the core of villi (red arrows) and pyknosis of their nuclei (arrowhead) (200 ×). **A6** (Infected combined IVM + atorvastatin-treated group) showing shortening and distortion of villi (black arrows) (200 ×).** A7** (Infected MSCs + IVM-treated group) showing marked improvement, only sloughing of small parts of the tip of some villi (black arrow). **A8** (Infected combined MSCs + atorvastatin-treated group) showing mild inflammatory cellular infiltrate appears (red arrows) (200 ×). **A9** (Infected combined MSCs + IVM + atorvastatin-treated group) appears near normal with no histopathological changes. **A10** (Infected combined MSCs + half dose of IVM + atorvastatin-treated) showing only mild inflammatory cells of the core of villi (red arrows) (200 ×)

Histopathological observations from non-infected control (Group A1) showed normal architecture of intestinal villi (Fig. 1A1). Controversially, infected non-treated control (Group A2) demonstrated dense inflammatory cellular infiltrate with sloughing and distortion of the villi, and sections in adult *T. spiralis* worms were noticed in the lumen and the core of villi (Fig. 1A2). On the other hand, variable degrees of regeneration and recovery of the previous histopathological changes were recorded among all infected treated mice (Groups A3–A10) (Fig. 1A3–A10). In this concern, dual therapies of IVM and atorvastatin (Group A6), MSCs and IVM (Group A7), and MSCs and atorvastatin (Group A8) showed more remarkable improvement than monotherapies administration (Groups A3–A5) (Fig. 1A3–A8). Moreover, a combined full dose of IVM and atorvastatin added to MSCs (Group A9) demonstrated the most prominent improvement with near normal villous appearance (Fig. 1A9), followed by infected mice treated with a combined half dose of IVM and atorvastatin added to MSCs (Group A10) that showed prolonged villi with mild vacuolations (Fig. 1A10).

#### Histopathological muscular phase results

**Fig. 2:**
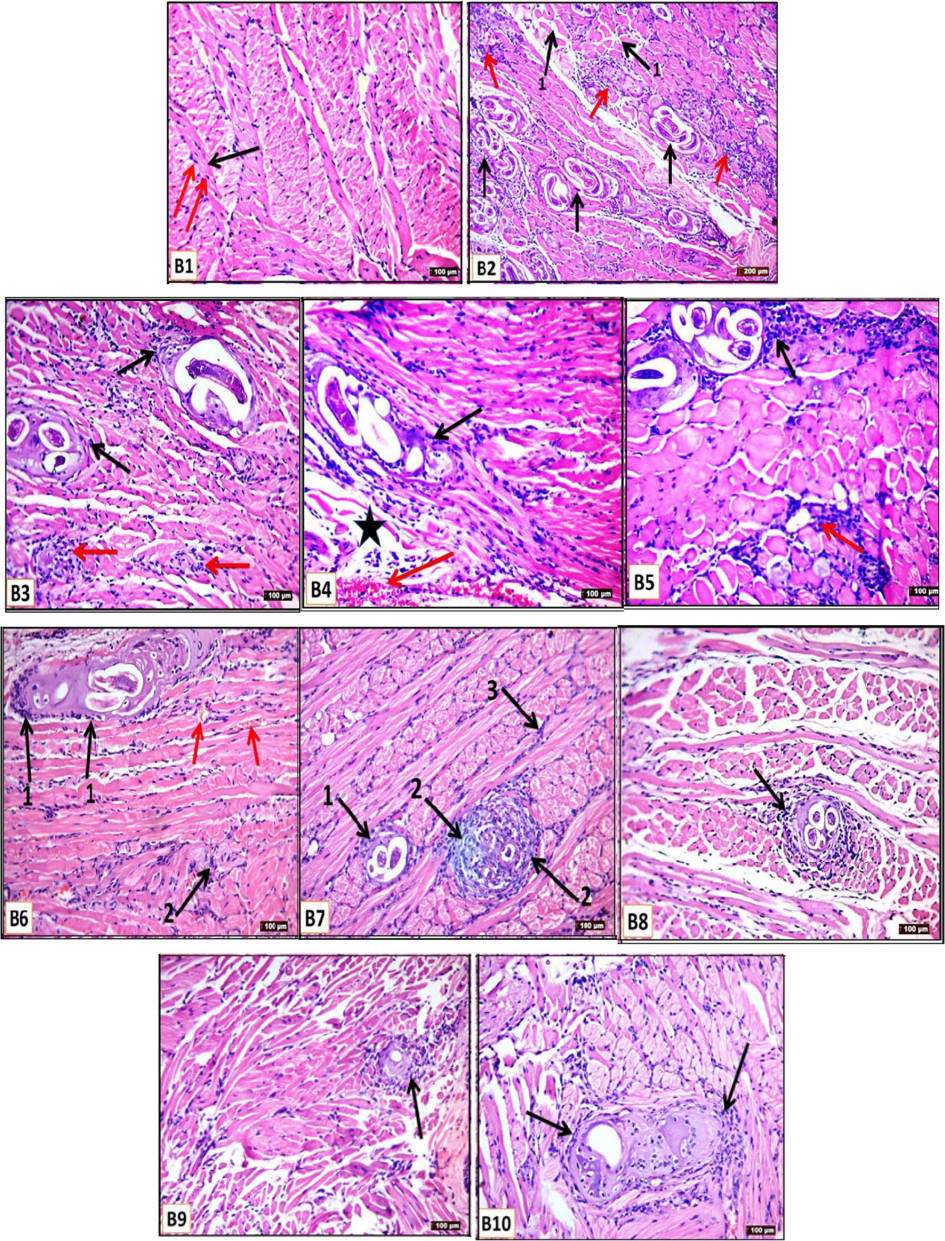
Sections of tongue muscle fibers stained with H&E from studied groups (**B1**–**B10**) on the day 35 PI; **B1** (non-infected control group) showing the normal architecture of muscle fibers which appear in a different direction have acidophilic cytoplasm (black arrow) and peripherally situated nuclei (red arrows) (200 ×). **B2** (infected non-treated control group) showing cut sections in encysted *T. spiralis* larvae within a nurse cell, collagenous capsule (black arrows), and dense inflammatory cellular infiltrations of the capsule (red arrows) with diffuse degenerative changes all over the muscle bundles. Widening between muscle fibers is also seen (1** →)** (100 ×). **B3** (Infected IVM-treated group) showing mild degenerated larvae with a moderate inflammatory cellular infiltrate of the capsule (black arrows) and between muscle fibers (red arrows**)** (200 ×). **B4** (Infected atorvastatin treated group) showing mild degenerated larvae with a moderate inflammatory cellular infiltrate of the capsule (black arrow), degenerative area (star), and congestion (red arrow) are also seen (200 ×). **B5** (Infected MSC-treated group) showing mild degenerated larvae with a moderate inflammatory cellular infiltrate of the capsule (black arrow) and between muscle fibers (red arrows) (200 ×). **B6** (Infected combined IVM + atorvastatin group) showing moderate degenerated larvae within a nurse cell with a mild inflammatory cellular infiltrate of the capsule (1 →) and in-between muscle fibers (2 →), minimal congestion (red arrows) are also seen (200 ×). **B7** (Infected combined MSCs + IVM group) moderate degenerated larvae with mild inflammatory cellular infiltrate of the capsule (1 →), other larvae show marked degeneration surrounded with moderate inflammatory cellular infiltration (2 →) with minimal inflammatory cellular infiltrate in between muscle fibers (3 →) (200 ×). **B8** (Infected combined MSCs + Atorvastatin group) moderate degenerated larvae with a moderate inflammatory cellular infiltrate of the capsule (black arrow) (200 ×). **B9** (Infected combined IVM + Atorvastatin + MSCs group) appears more or less as normal as the control group, with marked degenerated larvae and complete degeneration with a mild inflammatory cellular infiltrate of the capsule with apparently intact muscle fibers (black arrow) (200 ×). **B10** (Infected combined half dose of (IVM + Atorvastatin) + MSCs) showing marked degenerated larvae and complete degeneration with a mild inflammatory cellular infiltrate of the capsule (black arrow) (200 ×)

Histopathological examination of the tongue muscle of non-infected control (Group B1) showed normal architecture of the muscle fibers (Fig. 2B1). In contrast, infected non-treated control (Group B2) showed encysted *T. spiralis* larvae within a nurse cell and dense infiltration of the collagenous capsule by inflammatory cells with degenerative changes and widening in the surrounding muscle bundles (Fig. 2B2). Tissues from infected mice treated with the monotherapy of IVM, atorvastatin, or MSCs (Group B3–B5, respectively) revealed mild degeneration of larvae with a moderate inflammatory cellular infiltrate of the capsule (Fig. 2B3–B5). In addition, dual therapies of IVM and atorvastatin (Group B6), MSCs and IVM (Group B7), and MSCs and atorvastatin (Group B8) showed moderate degeneration of the larvae and obvious mild inflammatory reaction with intact muscle (Fig. 2B6–B8). At the same time, both combined full or half doses of IVM and atorvastatin added to MSCs (Group B9 and B10, respectively) showed marked reduction and disintegration of the deposited larvae with destruction of the capsules (Fig. 2B9 and B10, respectively).

### Biochemical studies results

#### Results of the inflammatory immune marker (IL-17) in serum

In both intestinal and muscular phases, the serum level of IL-17 was significantly increased (*p* < 0.001) with infected non-treated controls (Groups A2 and B2) compared to non-infected controls (A1 and B1, respectively). However, the level of IL-17 was significantly decreased among all infected treated mice (Groups A3–A10 and B3–B10) compared to infected non-treated controls (Groups A2 and B2), respectively. Additionally, the lowest level of IL-17 among treated mice was observed after administration of the combined full dose of IVM and atorvastatin added to MSCs (Groups A9 and B9), while atorvastatin monotherapy administration (Groups A4 and B5) showed the highest level (Table 3).

**Table 3:** Biochemical changes in inflammatory immune marker (IL-17) (pg/ml) in serum of all groups of intestinal and muscular phases

Groups	IL-17 (pg/ml)						
	Intestinal phase			Groups	Muscular phase		
	Mean ± SD	*GA1* (*p-*value)	*GA2* (*p-*value)		Mean ± SD	*GA1* (*p-*value)	*GA2* (*p-*value)
A1	149.33 ± 1.15		*P* < 0.001*	B1	154 ± 3.61		*P* < 0.001*
A2	193.33 ± 11.55	*P* < 0.001*		B2	248.33 ± 10.41	*P* < 0.001*	
A3	171.67 ± 2.89	*P* < 0.001*	*P* < 0.01*	B3	184.33 ± 4.04	*P* < 0.001*	*P* < 0.001*
A4	181 ± 6.56	*P* < 0.001*	*P* < 0.05*	B4	236.67 ± 17.56	*P* < 0.001*	*P* > 0.05
A5	173.33 ± 7.64	*P* < 0.001*	*P* < 0.01*	B5	194.33 ± 6.03	*P* < 0.001*	*P* < 0.001*
A6	167.33 ± 8.73	*P* < 0.001*	*P* < 0.01*	B6	176 ± 3.61	*P* < 0.001*	*P* < 0.001*
A7	159.33 ± 5.13	*P* < 0.001*	*P* < 0.001*	B7	172.67 ± 2.52	*P* < 0.001*	*P* < 0.001*
A8	170.67 ± 6.03	*P* < 0.001*	*P* < 0.01*	B8	186.33 ± 5.51	*P* < 0.001*	*P* < 0.001*
A9	155 ± 13.23	*P* > 0.05	*P* < 0.001*	B9	161.33 ± 3.21	*P* < 0.01*	*P* < 0.001*
A10	157.67 ± 2.52	*P* < 0.001*	*P* < 0.001*	B10	164 ± 5.29	*P* < 0.01*	*P* < 0.001*
ANOVA							
*F*-test	18.4	14.1	13.1		103.9	92.2	70.3
*P*-value	0.000*	0.000*	0.000*		0.000*	0.000*	0.000*

A1 and B1: non-infected control mice

A2 and B2: infected non-treated control mice

A3 and B3: infected IVM-treated mice

A4 and B4: infected atorvastatin-treated

A5 andB5: infected MSC-treated

A6 and B6: infected combined IVM + atorvastatin

A7 and B7: infected combined MSCs + IVM

A8 and B8: infected combined MSCs + atorvastatin

A9 and B9: infected combined MSCs + IVM + atorvastatin

A10 and B10: infected combined MSCs + half dose of (IVM + atorvastatin)

^*^P < 0.05

#### Results of the oxidative stress markers/antioxidant enzymes in tissue homogenate

Levels of lipid peroxidase, GSH, and SOD in the small intestinal and muscular tissue homogenates representing severe oxidative stress in infected non-treated controls (Groups A2 and B2) as they significantly increased (*p* < 0.001) compared to non-infected controls (Groups A1 and B1), respectively. However, among all treated mice (Groups A3–A10 and B3–B10), there is a significant decrease in the levels of the oxidative stress markers compared to infected non-treated control (Groups A2 and B2), respectively. Infected mice treated with the combined full dose of IVM and atorvastatin added to MSCs (Groups A9 and B9) showed the lowest oxidative stress markers/antioxidant enzymes (Tables 4 and 5).

**Table 4:** Biochemical changes in the oxidative stress markers/antioxidant enzymes in intestinal tissues

Intestinal groups	Lipid peroxidase (U/ml)			GSH (U/ml)			SOD (U/ml)		
	Mean ± SD	A1 (*p-*value)	A2 (*p-*value)	Mean ± SD	A1 (*p-*value)	A2 (*p-*value)	Mean ± SD	A1 (*p-*value)	A2 (*p-*value)
A1	2.33 ± 0.15		*P* < 0.001*	1.1 ± 0.1		*P* < 0.001*	18.1 ± 0.7		*P* < 0.001*
A2	8.5 ± 0.5	*P* < 0.001*		2.33 ± 0.15	*P* < 0.001*		38.7 ± 1.53	*P* < 0.001*	
A3	4.8 ± 0.3	*P* < 0.001*	*P* < 0.001*	1.9 ± 0.2	*P* < 0.001*	*P* < 0.01*	35.6 ± 0.57	*P* < 0.001*	*P* < 0.001*
A4	7.83 ± 0.2	*P* < 0.001*	*P* < 0.01*	2.03 ± 0.15	*P* < 0.001*	*P* < 0.01*	38.1 ± 1.03	*P* < 0.001*	*P* > 0.05
A5	5.93 ± 0.4	*P* < 0.001*	*P* < 0.001*	1.96 ± 0.05	*P* < 0.001*	*P* < 0.001*	36.5 ± 0.5	*P* < 0.001*	*P* < 0.01*
A6	4.63 ± 0.32	*P* < 0.001*	*P* < 0.001*	1.6 ± 0.1	*P* < 0.001*	*P* < 0.001*	26.2 ± 0.76	*P* < 0.001*	*P* < 0.001*
A7	4.47 ± 0.42	*P* < 0.001*	*P* < 0.01*	1.42 ± 0.07	*P* < 0.001*	*P* < 0.001*	23.8 ± 1.6	*P* < 0.001*	*P* < 0.001*
A8	5.2 ± 0.2	*P* < 0.001*	*P* < 0.01*	1.82 ± 0.07	*P* < 0.001*	*P* < 0.001*	27.7 ± 2.07	*P* < 0.001*	*P* < 0.001*
A9	3.53 ± 0.47	*P* < 0.001*	*P* < 0.001*	1.2 ± 0.1	*P* > 0.05	*P* < 0.001*	22.2 ± 0.76	*P* < 0.001*	*P* < 0.001*
A10	4 ± 0.2	*P* < 0.001*	*P* < 0.001*	1.34 ± 0.05	*P* < 0.001*	*P* < 0.001*	23.2 ± 3.01	*P* < 0.01*	*P* < 0.001*
ANOVA									
*F*-test	185.4	139.3	144.4	73.8	60.6	60.1	158.6	121.8	143.4
*P*-value	0.000*	0.000*	0.000*	0.000*	0.000*	0.000*	0.000*	0.000*	0.000*

A1: non-infected control mice

A2: infected non-treated control mice

A3: infected IVM-treated mice

A4: infected atorvastatin-treated

A5: infected MSC-treated

A6: infected combined IVM + atorvastatin

A7: infected combined MSCs + IVM

A8: infected combined MSCs + atorvastatin

A9: infected combined MSCs + IVM + atorvastatin

A10: infected combined MSCs + half dose of (IVM + atorvastatin)

^*^P < 0.05

**Table 5:** Biochemical changes in the oxidative stress markers/antioxidant enzymes in muscular tissues

Muscular groups	Lipid peroxidase (U/ml)			GSH (U/ml)			SOD (U/ml)		
	Mean ± SD	B1 (*P*-value)	B2 (*P*-value)	Mean ± SD	B1 (*P*-value)	B2 (*P*-value)	Mean ± SD	B1 (*P*-value)	B2 (*P*-value)
B1	11.17 ± 0.76		*P* < 0.001*	2.67 ± 0.21		*P* < 0.001*	2.6 ± 0.5		*P* < 0.001*
B2	22.33 ± 2.52	*P* < 0.001*		4.76 ± 0.25	*P* < 0.001*		9.3 ± 1.1	*P* < 0.001*	
B3	16.83 ± 1.04	*P* < 0.001*	*P* < 0.001*	4.13 ± 0.15	*P* < 0.001*	*P* < 0.01*	5.5 ± 0.7	*P* < 0.001*	*P* < 0.001*
B4	19.5 ± 0.5	*P* < 0.001*	*P* < 0.01*	4.63 ± 0.15	*P* < 0.001*	*P* < 0.01*	7.8 ± 0.25	*P* < 0.001*	*P* > 0.05
B5	18.57 ± 0.51	*P* < 0.001*	*P* < 0.001*	4.33 ± 0.15	*P* < 0.001*	*P* < 0.001*	7.2 ± 0.75	*P* < 0.001*	*P* < 0.01*
B6	15.5 ± 0.5	*P* < 0.001*	*P* < 0.001*	3.71 ± 0.9	*P* < 0.001*	*P* < 0.001*	5.6 ± 0.36	*P* < 0.001*	*P* < 0.001*
B7	14.67 ± 0.76	*P* < 0.001*	*P* < 0.01*	3.7 ± 0.11	*P* < 0.001*	*P* < 0.001*	4.8 ± 0.75	*P* < 0.001*	*P* < 0.001*
B8	16.5 ± 0.87	*P* < 0.001*	*P* < 0.01*	3.9 ± 0.1	*P* < 0.001*	*P* < 0.001*	6.7 ± 1.35	*P* < 0.001*	*P* < 0.001*
B9	12.17 ± 0.76	*P* < 0.001*	*P* < 0.001*	2.87 ± 0.15	*P* > 0.05	*P* < 0.001*	3.7 ± 0.49	*P* < 0.001*	*P* < 0.001*
B10	13.67 ± 0.76	*P* < 0.001*	*P* < 0.001*	3.2 ± 0.1	*P* < 0.001*	*P* < 0.001*	4.2 ± 0.8	*P* < 0.01*	*P* < 0.001*
ANOVA									
*F*-test	94.3	139.3	144.4	26.8	60.6	60.1	30.1	121.8	143.4
*P*-value	0.000*	0.000*	0.000*	0.000*	0.000*	0.000*	0.000*	0.000*	0.000*

B1: non-infected control mice

B2: infected non-treated control mice

B3: infected IVM-treated mice

B4: infected atorvastatin-treated

B5: infected MSC-treated

B6: infected combined IVM + atorvastatin

B7: infected combined MSCs + IVM

B8: infected combined MSCs + atorvastatin

B9: infected combined MSCs + IVM + atorvastatin

B10: infected combined MSCs + half dose of (IVM + atorvastatin)

^*^P < 0.05

### Markers of angiogenesis assay results

#### Immunohistochemical study results

**Fig. 3:**
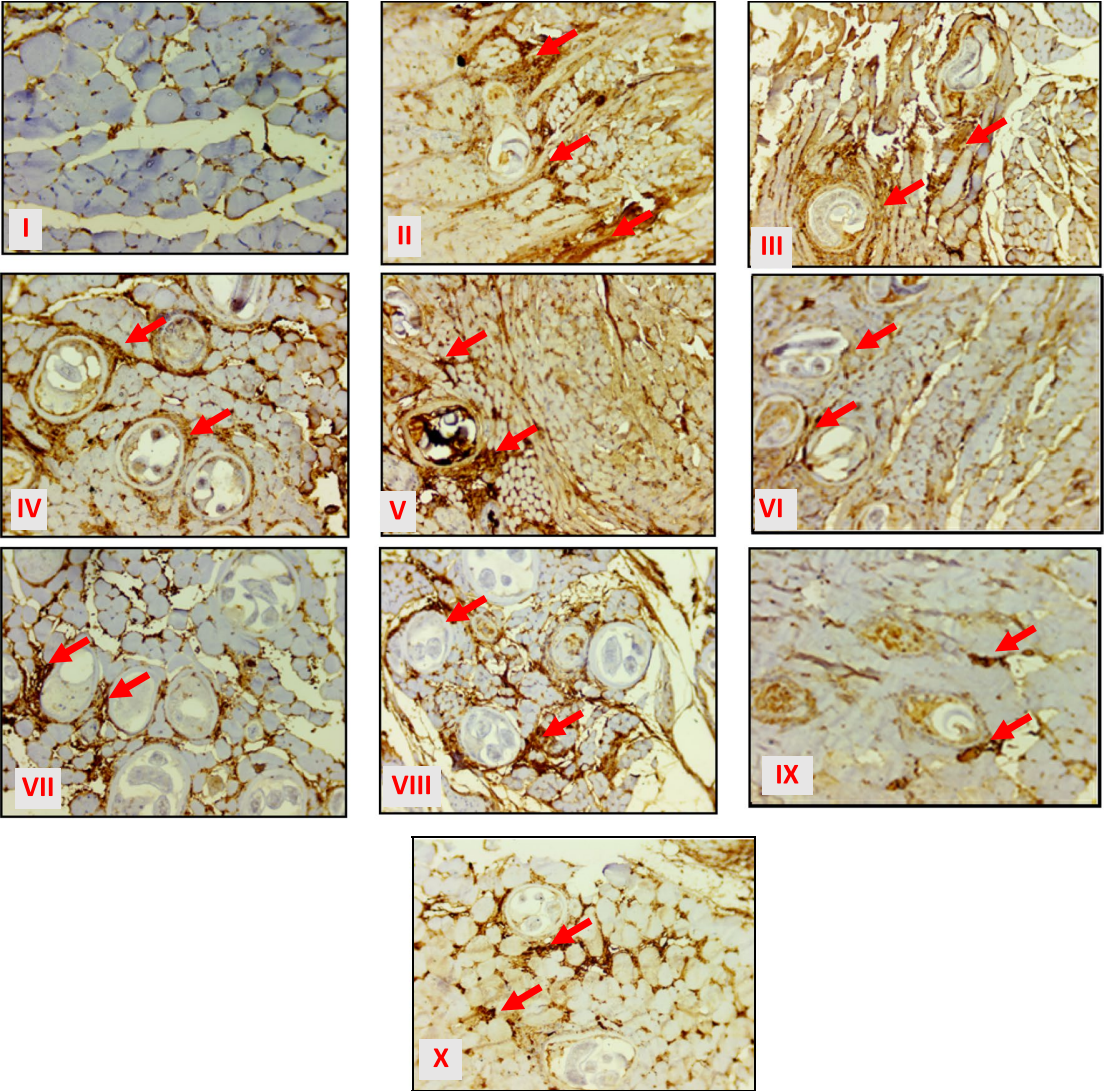
Immunohistochemical expression of CD 31 in different studied groups (groups; **B1**–**B10**) (200 ×). **I** Non-infected control (group B1) showing average lymphoid cells and normal vascularity. **II** Infected non-treated control (group B2) showing marked expression of inflammatory cells and strong MDV staining 45/field (red arrows). **III** Infected IVM-treated (group B3) showing moderate inflammation and strong MDV staining less than 30/field (red arrows). **IV** Infected atorvastatin-treated (group B4) showing moderate inflammation and strong MDV staining 35/field (red arrows). **V** Infected MSC-treated (group B5) showing moderate inflammation and strong MDV staining 40/field (strong MDV) (red arrows). **VI** Infected combined IVM + atorvastatin-treated (group B6) showing moderate inflammation and medium MDV staining 15/field (red arrows). **VII** Infected combined MSCs + IVM-treated (group B7) showing mild inflammation and medium MDV staining 18/field (red arrows). **VIII** Infected combined MSCs + atorvastatin-treated (group B8) showing moderate inflammation and medium MDV staining 20/field (red arrows). **IX** Infected combined MSCs + IVM + atorvastatin-treated (group B9) showing mild inflammation and weak MDV staining 8/field (red arrows). **X** Combined MSCs + half dose (IVM + atorvastatin)-treated (group B10) showing mild inflammation and weak MDV staining 9/field (red arrows)

Examination of the muscular specimens of the non-infected control (Group B1) showed average lymphoid cells and normal vascularity (Fig. 3I). In contrast, infected non-treated control (Group B2) revealed a dense presence of inflammatory cells and an average number of CD31 positive vessels/field with a strong score of MVD (Fig. 3II). However, sections from infected mice treated with the monotherapy of either IVM, atorvastatin, or MSCs (Groups B3, B4, and B5, respectively) reflected a varying decrease in the level of the inflammatory cells and the average number of CD31 positive vessels/field, but still scored as a strong MVD (Fig. 3III–V). On the other hand, dual therapies of IVM and atorvastatin (Group B6), MSCs and IVM (Group B7), and MSCs and atorvastatin (Group B8) showed moderate inflammation and an average number of CD31 positive vessels/field with a medium score of MVD (Fig. 3VI–VIII), while both of combined full or half doses of IVM and atorvastatin added to MSCs (Groups B9 and B10, respectively) showed a weak score of MVD (Fig. 3IX and X, respectively).

#### Molecular VEGF gene expression assay results

The VEGF gene expression level in the different groups' muscular tissues showed a significantly higher expression among all infected mice even after treatment (Groups B2–B10) compared to non-infected control mice (Group B1). Otherwise, treatment administration in infected mice resulted in a significant reduction in VEGF gene expression (Groups B3–B10) compared to infected non-treated control (Group B2). Furthermore, this reduction was more pronounced after administration of the combined full dose of IVM and atorvastatin added to MSCs (Group B9) (Table 6).

**Table 6:** The mean level of the VEGF gene expression among all groups of muscular phase by real-time PCR

VEGF gene expression			
Muscular groups	Mean ± SD	B1 (*p*-value)	B2 (*p*-value)
B1	0.014 ± 0.003		*P* < 0.001*
B2	0.96 ± 0.06	*P* < 0.001*	
B3	0.122 ± 0.003	*P* < 0.001*	*P* < 0.001*
B4	0.135 ± 0.004	*P* < 0.001*	*P* < 0.001*
B5	0.136 ± 0.004	*P* < 0.001*	*P* < 0.001*
B6	0.068 ± 0.003	*P* < 0.001*	*P* < 0.001*
B7	0.088 ± 0.007	*P* < 0.01*	*P* < 0.01*
B8	0.117 ± 0.002	*P* < 0.001*	*P* < 0.001*
B9	0.025 ± 0.002	*P* < 0.001*	*P* < 0.01*
B10	0.052 ± 0.002	*P* < 0.001*	*P* < 0.001*
ANOVA			
*F*-test	1092.59	1040.22	824.79
*P*-value	0.000*		

B1: non-infected control mice

B2: infected non-treated control mice

B3: infected IVM-treated mice

B4: infected atorvastatin-treated

B5: infected MSC-treated

B6: infected combined IVM + atorvastatin

B7: infected combined MSCs + IVM

B8: infected combined MSCs + atorvastatin

B9: infected combined MSCs + IVM + atorvastatin

B10: infected combined MSCs + half dose of (IVM + atorvastatin)

^*^P < 0.05

## Discussion

Trichinellosis is one of the serious and widely distributed parasitic diseases. It can induce an inflammatory reaction in the affected tissues that can be fatal if extended to vital organs such as the lung, heart, or CNS (Xu et al. 2019). Owing to these serious complications, this study aimed to find more effective therapeutic agents against trichinellosis than the currently available drugs.

In the current study, the administration of different treatment regimens revealed a significant reduction in the mean count of adults and larvae among the intestinal and muscular phase groups. IVM, atorvastatin, and MSCs monotherapies reduced the intestinal worms by 72.4%, 38.4%, and 53.02%, respectively, and the muscular larvae count by 78.8%, 45.3%, and 56.7%, respectively. It was noticed that IVM monotherapy was the most effective among monotherapy administrations. Similarly, Basyoni and El-Sabaa (2013) and Fadil et al. (2022) reported that IVM was highly effective against adult worms and encysted larvae in *T. spiralis-*infected mice. This can be attributed to the ability of IVM to modulate GABA-gated chloride channels of the nematode parasites than that of the infected host (Bai and Ogbourne 2016).

Additionally, the MSCs monotherapy proved its effectiveness against both intestinal adult worms and muscular larvae, and this disagrees with the results of Sarhan et al. (2021), who stated that injectable MSCs in the muscular phase showed more effectiveness than their oral administration in the intestinal phase of trichinellosis. This disagreement might be due to the different MSCs treatment schedules as in the current study; MSCs were administrated only by the intraperitoneal route in both phases of the disease. Consequently, the variation in results can be explained by the fact that oral administration of MSCs may decrease their effectiveness. Otherwise, these MSCs’ therapeutic effects may not directly target the parasite itself but may be due to their immunomodulatory and regenerative effects on the parasite sequels (Matthews and Noulin 2019).

Although atorvastatin administration showed the lowest reduction percentage among monotherapies in both intestinal and muscular phases, it showed a significant difference compared to infected non-treated controls. These results were highlighted by Othman et al. (2016), who mentioned that atorvastatin showed a significant anti-parasitic effect when administrated at a dose of 10 mg/kg/day for 1 week before infection of the mice by *T. spiralis* and continued till the 5th week PI. The mechanism of action of atorvastatin against *T. spiralis* parasite may be due to the ability of statins to disrupt cholesterol synthesis through inhibition of 3-hydroxy-3-methyl-glutaryl-coenzyme A (HMG-CoA) reductase that affects the parasite growth and survival as cholesterol is a major constituent of the parasite cell membrane and necessary for cell membrane organization and function (Parihar et al. 2019; Mangmee et al. 2020).

The present results revealed that dual therapies of IVM and atorvastatin, IVM and MSCs, and MSCs and atorvastatin reduced the intestinal worms by 74.6%, 80.2%, and 54.3%, respectively, and the muscular larvae count by 79.1%, 80.5%, and 67.6%, respectively. IVM and MSCs exerted the highest reduction among the dual therapies in both phases. Furthermore, combined therapy of full and half doses of IVM and atorvastatin added to MSCs reduced the intestinal worms by 87.6% and 82.3%, respectively, and the muscular larvae count by 83.95% and 82.2%, respectively. Interestingly, the triple therapy of IVM, atorvastatin, and MSCs displayed the highest synergistic effect, followed by dual therapies.

Regarding the histopathological results of the small intestine, infected non-treated control showed severe destruction of the villous architecture with sections of the adult worms in the lumen and inside the villous core as previously reported (Othman et al. 2016; Sarhan et al. 2021; El-Wakil et al. 2023; Fadil et al. 2022). Marked improvement of these histopathological changes was obtained after administering combined full or half doses of IVM and atorvastatin added to MSCs, followed by dual therapies and monotherapies.

On the other hand, the muscular sections of the infected non-treated control demonstrated a large number of encysted larvae with dense inflammatory infiltration to the surrounding capsule, as reported by previous studies (Basyoni and El-Sabaa 2013; Othman et al. 2016; Sarhan et al. 2021; Fadil et al. 2022; El-Wakil et al. 2023). Marked reduction and destruction of deposited larvae were observed after the administration of combined full or half doses of IVM and atorvastatin added to MSCs, followed by dual therapies, then monotherapies.

Some studies demonstrated that the administration of IVM (Basyoni and El-Sabaa 2013; Fadil et al. 2022), atorvastatin (Othman et al. 2016), and MSCs (Sarhan et al. 2021) monotherapies revealed some improvement of the histopathological changes caused by experimental trichinellosis in both intestinal and muscular phases. Similarly, our results recorded augmentation of these healing properties after dual and triple combinations.

IL-17 is a proinflammatory cytokine that is released by Th17 cells. It links innate and adaptive immune responses that play essential roles against *T. spiralis* infection (Song et al. 2019; Wang et al. 2020). The underlying tissue damage caused by trichinellosis is most probably mediated by the production of cytokines, as well as the release of reactive oxygen species (ROS) both by the host or the parasite itself as a defense mechanism. Thus, changes in the activity of some antioxidant enzymes such as lipid peroxidase, GSH, and SOD in the affected tissues may protect the host against the damage mediated by the released free radicals during oxidative stress (Othman et al. 2016; Gabrashanska et al. 2019).

Concerning the current biochemical results, the serum level of IL-17 was significantly increased in the infected non-treated controls of both the intestinal and muscular phases, indicating severe inflammatory reaction as a result of *T. spiralis* infection as reported by Song et al. (2019) and Wang et al. (2020). A significant decrease in the level of IL-17 was observed among the infected treated mice. It was observed that IVM exerted the most potent anti-inflammatory effect among the monotherapies, and this effect was potentiated after combinations with other agents, as in the triple therapies followed by the dual therapies. The anti-inflammatory effect of IVM was previously recorded by Arndts et al. (2015), who observed lower levels of IL-17 after repeated treatment by IVM in *Onchocerca volvulus*-infected patients. Additionally, Xie et al. (2023) demonstrated that IVM can protect against autoimmune encephalomyelitis by reducing IL-17 and other inflammatory mediators in experimentally infected mice. Similarly, atorvastatin revealed a potential immunomodulatory effect against rheumatoid arthritis by reducing IL-17A, TNF, and IL-6 levels (de Oliveira et al. 2020). Guan et al. (2022) reported that adipose stem cells suppress the IL-17 level in murine-induced atopic dermatitis.

Regarding the oxidative stress markers/antioxidant enzymes, the lipid peroxidase, GSH, and SOD levels in the intestinal and muscular tissue of the infected non-treated controls showed a significant increase, indicating severe oxidative stress during trichinellosis. This result concurs with Othman et al. (2016) and Gabrashanska et al. (2019). Significant lower levels of antioxidant enzymes were observed in tissues of infected mice after administration of various treatment regimens with sharp reduction after the triple combination of IVM, atorvastatin, and MSCs. In the same context, Soliman et al. (2013) observed lower activity of antioxidant enzymes such as SOD and protein carbonyl in *Trichinella-*infected mice after IVM treatment. The same was reported by Elmehy et al. (2021), who noticed that treatment with IVM added to niosomes and nano-crystals resulted in a significant reduction in antioxidant enzymes activities as lactate dehydrogenase and glutathione-S-transferase at both phases of trichinellosis. Moreover, Othman et al. (2016) noticed that atorvastatin reduced the levels of SOD and pAMPK in intestinal and muscular tissue homogenates in *T. spiralis-*infected mice. In contrast, da Costa Gonçalves et al. (2017) found that MSC transplantation increased the levels of GSH and SOD in a murine model of colitis that may act as an initial antioxidant defense to help repair the underlying inflamed colon.

Interestingly, the process of nurse cell complex formation in the muscular phase of trichinellosis is associated with overexpression of the VEGF gene as a major inducer of angiogenesis in the surrounding capsule (Othman et al. 2016; Elmehy et al. 2021). Therefore, angiogenesis can be evaluated by estimating the level of VEGF gene expression besides measuring the MVD through immunohistochemical staining of specific endothelial cell markers such as CD31 (Rayia et al. 2022). In the present study, muscular tissues of the infected non-treated control showed higher expression of CD31 immunohistochemical staining (strong MVD) and VEGF gene, denoting that underlying *T. spiralis* infection upregulates the angiogenesis process, and this confirms the previously reported results (Rayia et al. 2022; Fadil et al. 2022). Evaluation of CD31 immunohistochemical staining and VEGF gene among infected mice received different treatment regimens displayed lower expression denoting downregulation of the underlying angiogenic process. This anti-angiogenic action was most evident with triple therapies of IVM, atorvastatin, and MSCs followed by the dual therapies, while monotherapies exhibited a less prominent anti-angiogenic effect than that noticed with the other treatment regimens.

Fadil et al. (2022) recorded that administration of IVM alone in experimental trichinellosis resulted in lower expression of VEGF mRNA and CD31-positive stained vessels. The same was recorded by Elmehy et al. (2021), who noticed that niosomal IVM and nanocrystalline IVM showed enhanced anti-angiogenic effect as the level of VEGF measured by ELISA was significantly decreased in tissues of *Trichinella*–infected mice. Othman et al. (2016) mentioned that atorvastatin revealed lower immunohistochemical expression of VEGF in the skeletal muscles of *T. spiralis*-infected mice. Even in the current study, the MSCs showed mild over-expression of CD31 immunohistochemical staining and the VEGF gene among monotherapies. It can be clarified by the angiogenic properties of MSCs, as confirmed by Setiawan et al. (2022), who demonstrated that MSCs therapy enhanced the expression of VEGF and CD31 to improve the healing of duodenal perforation by the angiogenesis process.

## Conclusion

As per the findings of this study, IVM was superior to atorvastatin and MSCs against trichinellosis during the intestinal and muscular phases of the disease, and its anti-parasitic, anti-inflammatory, antioxidant, and anti-angiogenic effects were potentiated after added to atorvastatin and MSCs. Hence, atorvastatin and MSCs can be helpful as promising additive or synergistic therapeutic agents against trichinellosis.

## Data Availability

All used data during the study are included in this article.
